# Photodynamic Bone Stabilization in Pathologic Humeral Fractures: Implant Safety, Pain Relief and Functional Outcomes

**DOI:** 10.3390/cancers18203285

**Published:** 2026-10-12

**Authors:** Julian Haupt, Beatrice Jung, George Farah, Philipp Drees, Erol Gercek, Saskia Sachsenmaier, Matthias Ring, Frank Traub

**Affiliations:** 1 Department for Trauma Surgery and Orthopaedics, Reconstructive and Septic Surgery, and Sports Traumatology, German Armed Forces Hospital Ulm, 89081 Ulm, Germany; matthiasring@bundeswehr.org; 2 Department of Orthopedics and Traumatology, University Medical Center Mainz, 55131 Mainz, Germanyfrank.traub@unimedizin-mainz.de (F.T.); 3 Department of Orthopedic Surgery, University Hospital of Tübingen, 72076 Tübingen, Germany

**Keywords:** pathologic humeral fracture, bone metastases, multiple myeloma, photodynamic intramedullary bone stabilization, palliative orthopedic oncology, skeletal-related events

## Abstract

**Background:** Pathologic fractures of the humerus due to metastatic disease cause severe pain, loss of function and reduced quality of life. Surgical treatment is predominantly palliative and aims to achieve rapid pain relief, early functional recovery and minimal treatment-related morbidity. The photodynamic bone stabilization system (IlluminOss^®^) is a minimally invasive, radiolucent, intramedullary device that may address these needs. **Methods:** This retrospective analysis of prospectively collected data from two centers included 51 patients with 52 pathologic humeral lesions (completed or impending fractures) treated with photodynamic intramedullary stabilization between 2013 and 2025. Baseline variables comprised demographics, Eastern Cooperative Oncology Group (ECOG) performance status, comorbidities, pain (numeric rating scale, NRS) and function (Musculoskeletal Tumor Society Upper Extremity Score, MSTS-UE; Toronto Extremity Salvage Score, TESS). Lesion characteristics, operative details, in-hospital course, follow-up outcomes (TESS, patient satisfaction/quality of life) and long-term endpoints (survival, complications) were recorded. **Results:** The study cohort (25 women, 26 men; mean age 68.5 years) represented a highly palliative population with advanced, disseminated malignant disease. Of the 52 humeral lesions, 35 (67.3%) presented as pathological fractures, whereas 17 lesions (32.7%) were classified as impending fractures due to extensive osteolysis. Among the impending fractures, Mirels scores ranged from 8 to 10, with three lesions scoring 8, eight lesions scoring 9, and six lesions scoring 10. ECOG performance status was 0–2 in 75.0% and 3–4 in 25.0% of patients. Preoperative pain was high (mean NRS 7.1) and function markedly reduced (median MSTS-UE 9.5/30; median TESS 36.5/100). Postoperatively, pain improved significantly from NRS 7.1 to 2.0 (*p* < 0.001). MSTS-UE increased from a median of 9.5 to 20 (*p* < 0.001). Follow-up showed further functional improvement with an increased TESS of 36.5 to 61 (*p* < 0.001), high patient satisfaction and subjective improvement in quality of life. Kaplan–Meier analysis (*n* = 44) demonstrated a median survival of 6 months for ECOG 0–2 and 3 months for ECOG 3–4. Implant failure (Henderson type III) occurred four times (7.7%) and was successfully revised with plate and/or screw osteosynthesis or diaphyseal replacement. **Conclusions:** Photodynamic intramedullary stabilization provides effective treatment for selected pathologic humeral lesions with low implant failure rate, offering rapid pain relief, functional improvement and low perioperative morbidity. Implant failure in complex fracture morphologies highlights the need for morphology-adapted indication and consideration of supplemental fixation in high-risk cases.

## Simple Summary

Pathologic fractures of the humerus most commonly arise from bone metastases and are associated with severe pain, functional loss and reduced quality of life. Many patients have limited life expectancy, so surgical treatment is primarily palliative and aims at rapid pain relief, restoration of basic function, low procedural morbidity and uninterrupted continuation of oncologic therapy. The photodynamic bone stabilization system (IlluminOss^®^) is a minimally invasive, radiolucent intramedullary implant that hardens in situ after light activation. It promises short operative times, limited soft-tissue trauma, early mobilization and artifact-free postoperative imaging and radiotherapy planning. In this study, we analyzed 51 patients with 52 pathologic humeral fractures or impending fractures treated with photodynamic intramedullary stabilization. We observed marked pain reduction, substantial functional improvement, short postoperative hospital stays and a low complication rate. Four cases of implant failure occurred, highlighting both the need for careful indication, particularly in complete distal fractures or extensive metaphyseal destruction while demonstrating a low failure rate. Our results support photodynamic stabilization as an effective palliative option for humeral fractures and impending fractures, while emphasizing the importance of morphology-adapted implant selection and multidisciplinary care pathways.

## 1. Introduction

Pathologic fractures of the humerus are a common manifestation of advanced malignant disease, most frequently caused by bone metastases of breast cancer, renal cell carcinoma, lung cancer, multiple myeloma, oropharyngeal carcinoma and gastrointestinal tumors [1,2,3,4]. They are associated with pronounced pain, loss of upper limb function and substantial deterioration of quality of life [5]. In contrast to traumatic fractures, treatment decisions must integrate oncologic prognosis, systemic disease burden and the palliative goals of care. For most patients, surgical intervention is not curative but aims to achieve rapid pain relief, restore basic function, minimize treatment-related morbidity and allow timely continuation of systemic therapy or radiotherapy [6,7,8]. Pathologic fractures represent a major skeletal-related event and have additionally been associated with reduced survival in patients with malignant bone disease [9].

The humerus represents one of the most frequent sites of metastatic involvement in the appendicular skeleton [1]. Humeral fractures significantly impair independence, as even limited arm function is essential for daily activities, self-care and mobility aids. Surgical stabilization is therefore often indicated, particularly in patients with reasonable functional status or an impending fracture at high risk of progression [7,8]. However, optimal fixation remains challenging because metastatic bone is mechanically compromised, fracture patterns are heterogeneous and patients frequently present with comorbidities and limited physiological reserve.

Conventional surgical options include plate osteosynthesis, intramedullary nailing and endoprosthetic reconstruction, with technique selection largely guided by fracture location, extent of bone destruction and expected survival [10,11,12]. While these methods are effective, they may be associated with substantial soft-tissue trauma, longer operative times and metal-related imaging artifacts that can complicate postoperative radiotherapy planning [10,13,14].

Photodynamic intramedullary bone stabilization has emerged as a minimally invasive alternative [13,15,16,17,18]. Using a balloon catheter filled with a light-curable polymer, the implant conforms to the intramedullary canal and hardens in situ, providing immediate internal stabilization. Potential advantages include limited surgical exposure, reduced blood loss, early mobilization and complete radiolucency, facilitating postoperative imaging and adjuvant radiotherapy [15,16,17,18,19]. Clinical reports suggest favorable outcomes in selected pathologic and osteoporotic fractures [15,16,17,18]. However, data on humeral lesions remain limited and concerns persist regarding mechanical strength in complex fracture morphologies.

The present retrospective analysis of prospectively collected data from two centers aims to evaluate the clinical outcomes of photodynamic intramedullary stabilization in pathologic humeral fractures and impending fractures. Specifically, we assessed pain relief, functional recovery, perioperative parameters, complications and survival, with particular attention to morphology-dependent limitations and the prognostic relevance of performance status. By doing so, we seek to better define the role of this technique within the palliative surgical management of humeral metastatic disease.

### Objective

The present study aimed to analyze (1) patient and fracture characteristics in pathologic humeral lesions treated with photodynamic intramedullary stabilization, (2) operative and short-term outcomes, (3) long-term functional outcome, quality of life, survival and (4) complications, including implant behavior and failure modes.

## 2. Materials and Methods

### 2.1. Study Design and Setting

This retrospective analysis of prospectively collected data included patients treated with photodynamic intramedullary stabilization of a pathologic humeral lesion consecutively at two institutions. The patients were treated at the Department of Orthopedic Surgery at the University Hospital Tübingen between 2013 and 2019 and subsequently at the Department of Orthopedics and Traumatology at the University Medical Center of the Johannes Gutenberg University Mainz between 2019 and 2025. Patients with a pathologic humeral fracture or an impending fracture due to metastatic bone disease were treated surgically using the IlluminOss^®^ Photodynamic Bone Stabilization System. All patients with 18 years of age or older, that received treatment with intramedullary, photodynamic bone stabilization for impending or complete pathologic fractures were screened. Patients with a fracture localization other than the humerus were excluded as well as patients with a non-malignant underlying disease, e.g., osteoporosis or hepatic osteodystrophy. Furthermore, due to the inherent surgical concept no primary malignant bone tumors were included. After screening according to predefined inclusion and exclusion criteria, 51 patients with 52 pathologic humeral lesions were included in the final analysis. The indication for photodynamic stabilization was primarily based on the local fracture morphology and the clinical assessment of the treating surgical team.

### 2.2. Collected Variables and Outcome Measures

Clinical, oncologic and surgical data were extracted from electronic medical records of both institutions. Baseline variables included age, sex, primary tumor entity, ECOG performance status, presenting symptoms and preoperative pain intensity measured using the numeric rating scale (NRS, 0–10). Preoperative function was assessed using the Musculoskeletal Tumor Society Upper Extremity Score (MSTS-UE, range 0–30) and the Toronto Extremity Salvage Score (TESS, range 0–100), where available [20,21]. Lesion characteristics comprised fracture status (completed vs. impending), anatomical location within the humerus (proximal, diaphyseal, distal) and presence of additional skeletal or visceral metastases. Lesions were classified as completed pathologic fractures when a pathological fracture was present on imaging. Lesions without a completed fracture but with extensive osteolytic destruction and a high risk of fracture were classified as impending fractures with the corresponding Mirels’ score [22,23]. Perioperative parameters included time from symptom onset to surgery, operative duration, need for additional osteosynthesis and postoperative mobilization regimen. Short-term outcomes included postoperative pain (NRS), functional outcome (MSTS-UE), postoperative length of hospital stay, red blood cell transfusion and postoperative infection. Follow-up outcomes included patient-reported functional recovery (TESS), patient satisfaction and subjective improvement in quality of life assessed using a 6-point Likert scale ranging from 1 (very dissatisfied) to 6 (very satisfied). Long-term outcomes comprised implant failure classified according to the Henderson classification, rate and timing of postoperative radiation therapy and overall survival. Due to incomplete documentation and heterogeneous follow-up, the number of evaluable patients varied between outcome measures. Only patients with available paired preoperative and postoperative assessments were included in the respective analyses.

### 2.3. Statistical Analysis

Data were collected using Microsoft^®^ Excel for Mac (version 16.78). Descriptive statistics were used to summarize patient, lesion, operative, in-hospital, follow-up and long-term variables. Comparisons between paired preoperative and postoperative NRS, MSTS-UE, and TESS scores were performed using paired t-tests. Only patients with available paired preoperative and postoperative assessments were included in the respective analyses. Kaplan–Meier survival analysis was performed using IBM SPSS Statistics (version 29.0.1.0). Comparisons between preoperative and postoperative outcomes were performed using GraphPad Prism (version 10.4.1). A *p*-value < 0.05 was considered statistically significant.

## 3. Results

A total of 51 patients with 52 pathologic humeral lesions treated with photodynamic intramedullary stabilization were included. Due to incomplete documentation in some variables, the number of evaluable patients is reported per analysis where applicable.

### 3.1. Patient Characteristics

The cohort comprised 25 women and 26 men with a mean of 68.5 years. All patients presented with pain as the main symptom. Additional symptoms such as swelling, visible deformity, hematoma or sensory deficits were documented but occurred less frequently. Preoperative function was substantially impaired. The median preoperative TESS was 36.5/100 and the median MSTS-UE was 9.5/30, indicating considerable functional limitation of the affected arm. Preoperative pain was high, with a mean NRS of 7.1. The ECOG performance status was available for 48 patients: 75.0% were ECOG 0–2 and 25.0% ECOG 3–4. Comorbidities such as diabetes mellitus and nicotine abuse were documented in 25.0% of patients each.

### 3.2. Lesion and Fracture Characteristics

Lesion characteristics reflected the typical spectrum of metastatic humeral disease. Of the 52 humeral lesions, 35 (67.3%) were completed pathologic fractures, while 17 (32.7%) were impending fractures due to extensive osteolysis. Among the impending fractures, Mirel’ scores ranged from 8 to 10, with three lesions scoring 8, eight lesions scoring 9, and six lesions scoring 10. The majority of lesions were located in the diaphysis (26/52, 50.0%), followed by the proximal humerus (24/52, 46.2%). Only two lesions (3.8%) involved the distal humerus, reflecting both the rarity and technical complexity of distal pathologic humeral fractures. There was a slight predominance of right-sided lesions (55.8%), although handedness was not systematically recorded. All lesions were secondary to malignant disease. The most common primary malignancies were multiple myeloma (28.8%), bronchial carcinoma (25.5%) and breast carcinoma (11.5%) with less frequent contributions from renal cell (7.7%) and prostate carcinoma (5.8%) and other primaries. In addition to the primary lesion in the humerus, further bone metastases were documented in 33 of the 52 cases. The most frequent extraskeletal metastasis sites were the lymph nodes (12 cases) and the lungs (9 cases).

### 3.3. Operative Parameters

The median time from onset of symptoms to operation was 27 days, indicating a clinically significant delay between first symptoms and definitive surgical stabilization. Median operative time was 67.5 min (mean 82.3 min). When no additional osteosynthesis was required, median operative duration decreased to 62 min. Additional osteosynthesis (screws or plates) was selectively used, primarily in complex metaphyseal or distal fractures, or where extensive curettage created large defects. Postoperative regimens reflected the confidence in implant stability: 73.0% of patients were mobilized with free functional use of the arm, 25.0% were allowed pain-adapted loading and only 2.0% required temporary immobilization in an upper-arm cast.

### 3.4. In-Hospital Outcomes

Short-term postoperative outcomes demonstrated substantial early benefits. Postoperative pain decreased significantly from a preoperative mean NRS of 7.1 to 2.0 postoperatively (*p* < 0.001) (Figure 1). This reduction indicates a clinically meaningful improvement in pain control and was observed across fracture types.

The MSTS-UE score improved from a median of 9.5 preoperatively to 20 postoperatively (*p* < 0.001), indicating a marked functional gain (Figure 2). Many patients could again use the arm for basic activities of daily living within days after surgery.

Median hospital stay was 3 days. Transfusion of red blood cell concentrates was rare (two cases, due to pre-existing anemia or anticoagulants). One case of postoperative surgical site infection was documented in the cohort and successfully treated with antibiotics with parallel revision due to implant failure. These findings align with the minimally invasive nature and limited soft-tissue trauma of the procedure.

### 3.5. Follow-Up Outcomes

Follow-up intervals varied substantially, reflecting differences in prognosis and oncologic trajectories. Postoperative radiotherapy was frequently recommended and often administered. The timing from surgery to radiotherapy initiation spanned a wide range due to interdisciplinary planning and scheduling constraints. Systemic therapies (chemotherapy, targeted or immunotherapy) were given according to tumor type and disease stage, either preoperatively, postoperatively or both. Functional outcomes continued to improve during follow-up. TESS scores increased over time from 36.5 to 61 (*p* < 0.001), reflecting progressive recovery of arm function (Figure 3).

In an exploratory, non-validated patient-reported assessment, 31 of 39 patients (79.4%) reported a subjective improvement in their perceived quality of life following the procedure. In addition, the postoperative Likert-scale assessment yielded a median score of 4 (mean 4.25; range 3–6; *n* = 28). As this assessment was not based on a validated quality-of-life instrument, these findings should be considered descriptive and exploratory.

### 3.6. Long-Term Outcomes

Long-term outcomes encompassed survival and implant-related events.

Kaplan–Meier analysis (*n* = 44 patients with complete data) demonstrated a median survival of 6 months. Coincidentally, the survival was also 6 months in patients with ECOG 0–2, compared with 3 months in those with ECOG 3–4. The difference did not reach statistical significance (*p* = 0.0933), but the trend supports the potential role of ECOG as a prognostic marker. Overall, the survival profile reflects a typical palliative metastatic population. Postoperative radiation therapy was recommended by the multidisciplinary tumor board in 67.3% of cases. In 80.0% of these cases, postoperative eligibility for radiation therapy was documented at discharge. Four cases (7.7%) of implant failure were observed, all classified as Henderson type III (structural failure). In all four cases, structural failure was characterized by fracture of the bone and the photodynamic implant. Implant failure occurred at 9, 62, 75 and 409 days after index surgery, respectively. In all cases, implant failure occurred during follow-up and required revision surgery. Three cases were treated with plate and/or screw osteosynthesis, whereas one case required diaphyseal replacement. An overview of the characteristics of the four implant failure cases is provided in Table 1. Following one of the revision surgeries, a wound infection was observed and was successfully treated with antibiotic therapy.

## 4. Discussion

### 4.1. Principal Findings

In this study, photodynamic intramedullary stabilization provided rapid and clinically meaningful pain relief, substantial functional improvement and low perioperative morbidity in patients with pathologic humeral lesions. Postoperative pain decreased markedly, functional scores improved significantly and postoperative hospitalization was short. Implant-related complications were infrequent but occurred in complex fracture morphologies, underscoring the importance of careful indication and morphology-adapted stabilization strategies.

These findings support photodynamic intramedullary stabilization as an effective palliative option for selected humeral lesions, particularly diaphyseal impending fractures, while highlighting clear mechanical limitations in advanced or distal fracture patterns.

### 4.2. Pain Relief and Functional Recovery in a Palliative Setting

Pain relief represents the most relevant patient-centered endpoint in palliative orthopedic oncology. In the present cohort, pain intensity decreased from a mean NRS of 7.1 preoperatively to 2.0 postoperatively, indicating rapid and clinically relevant analgesia. This magnitude of pain reduction is consistent with previously published series on photodynamic stabilization of humeral metastases and reflects the immediate biomechanical effect of intramedullary stabilization on tumor-related mechanical pain [16,24].

Functional recovery followed a similar pattern. MSTS-UE improved from a median of 9.5 to 20, corresponding to a shift from severe impairment to functional use of the affected arm in daily activities. Early mobilization was feasible in the majority of patients, facilitated by the minimally invasive nature of the procedure and limited soft-tissue trauma. These outcomes are particularly relevant in patients with limited life expectancy, where prolonged immobilization or delayed recovery may negate potential benefits of surgical intervention. Furthermore, TESS showed a significant increase perioperatively from 36.5 to 61. Previous studies report physician-reported outcomes like MSTS-UE. This study emphasizes the importance of patient-reported outcomes (PRO) like TESS and is able to demonstrate significant improvement for patients when treated with photodynamic bone stabilization. Furthermore, TESS offers more specific investigation into PRO of the upper extremity than other generalized PRO like QLQ-BM22.

Validated quality-of-life instruments were not systematically applied. Therefore, the reported subjective improvement in perceived quality of life and the Likert-scale ratings should be regarded as exploratory, non-validated patient-reported observations and interpreted with caution. Nevertheless, improvements in functional scores and high patient satisfaction suggest that photodynamic stabilization may positively affect key dimensions of daily living in this vulnerable population.

### 4.3. Procedural Efficiency, Safety and Oncologic Pathways

Procedural efficiency is a critical consideration in patients with advanced malignancy, who often present with limited physiological reserve and high perioperative risk. A key technical advantage of photodynamic stabilization is the minimally invasive approach, typically requiring only a small incision of approximately 3–5 cm. In cases without supplementary fixation, the median operative time was 62 min, compared with 99 min in patients requiring supplementary fixation. The overall median operative time was 67.5 min. This distinction is important, as supplementary fixation reflects more complex local situations rather than the procedural burden of photodynamic stabilization itself. Blood transfusion was rare, and no primary postoperative infections were observed. Compared with open plate fixation or conventional intramedullary nailing, photodynamic stabilization may therefore reduce surgical exposure and soft-tissue trauma, which is particularly relevant in this highly palliative cohort [25,26]. In addition, the implant’s radiolucency facilitates postoperative imaging and radiotherapy planning, supporting integration into multimodal oncologic care [14,16,19]. In most patients for whom postoperative radiotherapy was recommended, eligibility for radiation treatment was already documented at discharge, suggesting that this minimally invasive approach may help avoid unnecessary delays in subsequent cancer-directed therapy.

ECOG performance status emerged as a prognostic indicator in this cohort. Patients with ECOG 0–2 demonstrated longer median survival compared with those classified as ECOG 3–4. Although the survival difference did not reach statistical significance, likely due to limited sample size, the trend aligns with extensive oncologic literature [27,28]. ECOG performance status should therefore be considered as one component of surgical decision-making.

### 4.4. Implant Failure and Indication Limits of Photodynamic Monotherapy

Despite overall favorable outcomes, implant-related failure occurred in four cases and was classified as Henderson type III structural failure. Three of the four structural failures occurred relatively early, at 9, 62, and 75 days after the index procedure, without a relevant preceding trauma or following only an inadequate trauma. In contrast, the late failure at 409 days occurred following a low-level fall onto the affected arm, suggesting that trauma may have contributed to this late failure. All cases required revision surgery with plate and/or screw osteosynthesis or diaphyseal replacement. These failures highlight the mechanical limits of photodynamic intramedullary stabilization, particularly in complex fracture morphologies [18,24,29,30]. Evidence from the literature suggests that complete fractures, distal humeral involvement, extensive metaphyseal destruction and long defect lengths impose increased mechanical demands on polymer-based implants. In such scenarios, standalone photodynamic stabilization may be insufficient [18,24,29,30]. Nonetheless, our study reports an overall low implant failure rate of 7.7% compared to previous studies reporting around 15% failure rate [13,24]. Our findings support a low threshold for supplementary fixation or alternative stabilization methods in high-risk morphologies. Clear indication limits and standardized decision algorithms would likely reduce implant failure risk.

### 4.5. Strengths and Limitations

Strengths of this study include the focus on a clinically relevant palliative cohort, the use of validated functional measures (MSTS-UE), incorporation of ECOG performance status as a key prognostic variable and the inclusion of both short-term and longer-term outcomes. Furthermore, this study includes PRO specific to the upper extremity.

This study has several limitations inherent to its retrospective analysis. The absence of a control group precludes direct comparison with alternative fixation methods; the sample size was limited and there was incomplete data for some variables. The absence of a control group represents a major limitation of this study and precludes causal attribution of the observed improvements specifically to photodynamic stabilization. Although significant improvements in pain and functional outcome measures were observed following surgery, these changes should be interpreted as clinical outcomes within a multimodal oncologic treatment setting rather than as treatment-specific effects of the photodynamic implant. Potential contributing factors include the general effect of surgical stabilization, concomitant radiotherapy or systemic oncologic treatment, postoperative rehabilitation, the natural course of the pathologic lesion, placebo effects, and regression to the mean. The relative contribution of these factors cannot be determined from the present study design. Accordingly, controlled prospective studies are required to determine the treatment-specific effect of photodynamic stabilization relative to established surgical techniques and, where clinically appropriate, non-operative treatment. A further limitation is the incomplete follow-up inherent to this highly palliative cohort. In several patients, continued follow-up at the treating orthopedic oncology center was no longer clinically necessary after postoperative recovery and transition to local or systemic oncologic care. This may have introduced selection bias; the direction and magnitude of any potential bias cannot be reliably determined. The exact implant survival until death could not be reliably assessed in all cases. Furthermore, the limited survival of many patients may have reduced the observation period during which late mechanical implant failure could become clinically apparent. Therefore, the absence of implant failure during a short follow-up period should not be interpreted as evidence of long-term implant durability. Late mechanical complications may remain undetected in patients who died or were subsequently followed outside our orthopedic oncology centers. Functional and pain assessments were not performed at standardized postoperative time points, which substantially limits the interpretation of temporal recovery trajectories. Accordingly, changes in outcome scores should be interpreted as differences between baseline and available follow-up assessments rather than as a standardized longitudinal recovery course.

Nevertheless, the convergence of our findings with those from external cohorts supports the validity of the observed patterns.

### 4.6. Clinical Implications and Future Research

The results of this study support a morphology-adapted, patient-centered approach to surgical stabilization of pathologic humeral lesions. Photodynamic intramedullary stabilization appears particularly suitable for diaphyseal fractures in patients with preserved functional status, offering rapid pain relief and early mobilization with low perioperative burden.

Future research should focus on prospective comparative studies evaluating photodynamic stabilization against established techniques, stratified by fracture morphology and performance status. Standardized pain and functional assessment time points, biomechanical modeling and integration of PRO measures are needed to refine indication criteria and optimize treatment algorithms.

## 5. Conclusions

Photodynamic intramedullary stabilization represents an effective minimally invasive option for the treatment of pathologic humeral lesions. In this cohort, the technique provided rapid and clinically meaningful pain relief, significant functional improvement, short postoperative hospitalization and a low rate of perioperative complications.

However, implant performance proved to be strongly morphology-dependent. While diaphyseal lesions and impending fractures were reliably stabilized using photodynamic monotherapy, structural implant failure occurred in complex fracture patterns, underscoring the need for careful patient selection and a low threshold for supplemental fixation or alternative stabilization strategies in high-risk configurations. Performance status, particularly ECOG classification, remains a key factor in surgical decision-making and outcome stratification.

Overall, photodynamic intramedullary stabilization should be considered a valuable component within a morphology- and prognosis-adapted surgical algorithm for pathologic humeral lesions. Further prospective comparative studies are warranted to refine indication criteria and to better define its role relative to established fixation techniques.

## Figures and Tables

**Figure 1:**
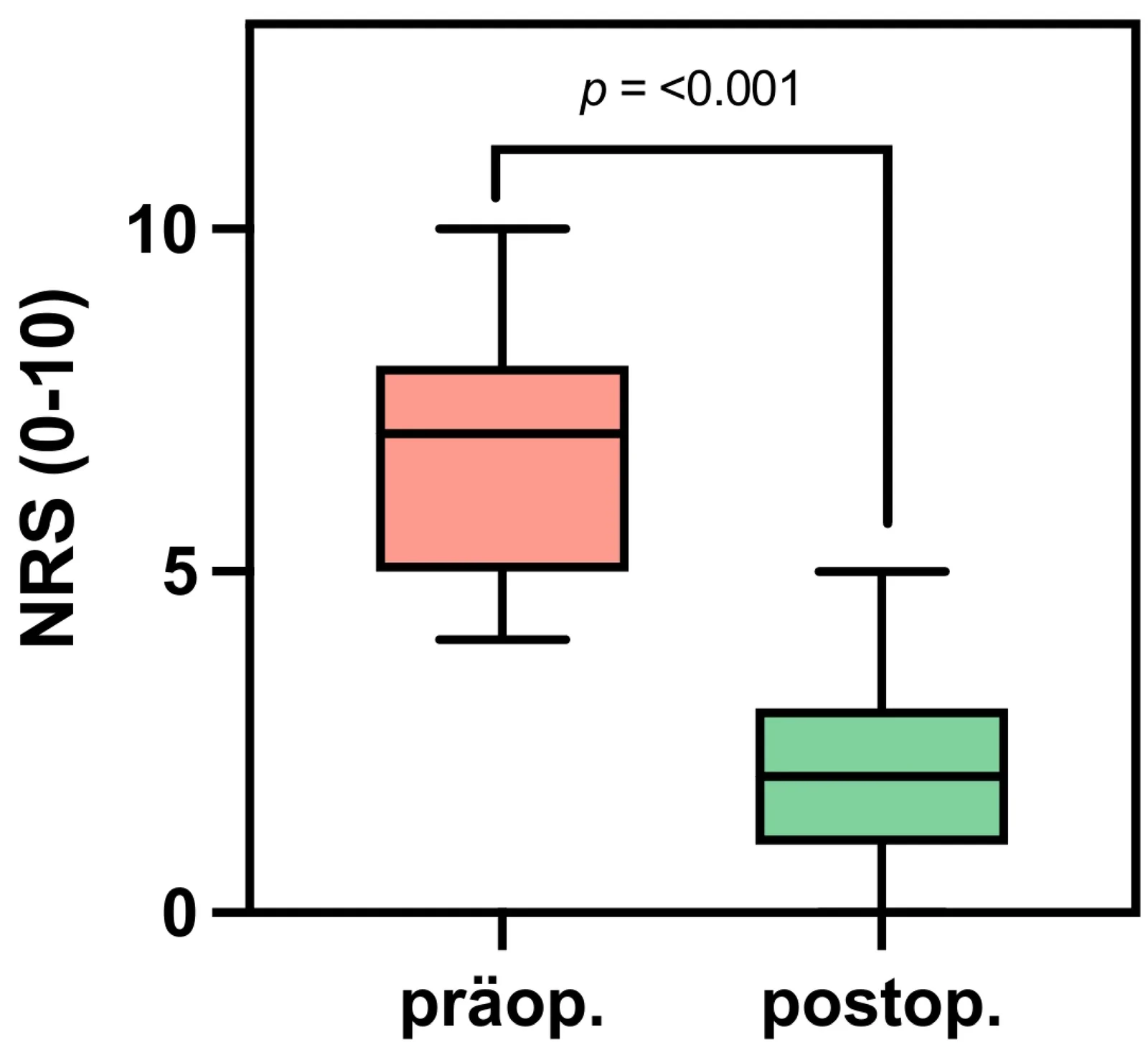
Comparison of preoperative and postoperative values on the NRS after photodynamic stabilization in patients with pathologic humerus fractures, paired *t*-test, *n* = 29.

**Figure 2:**
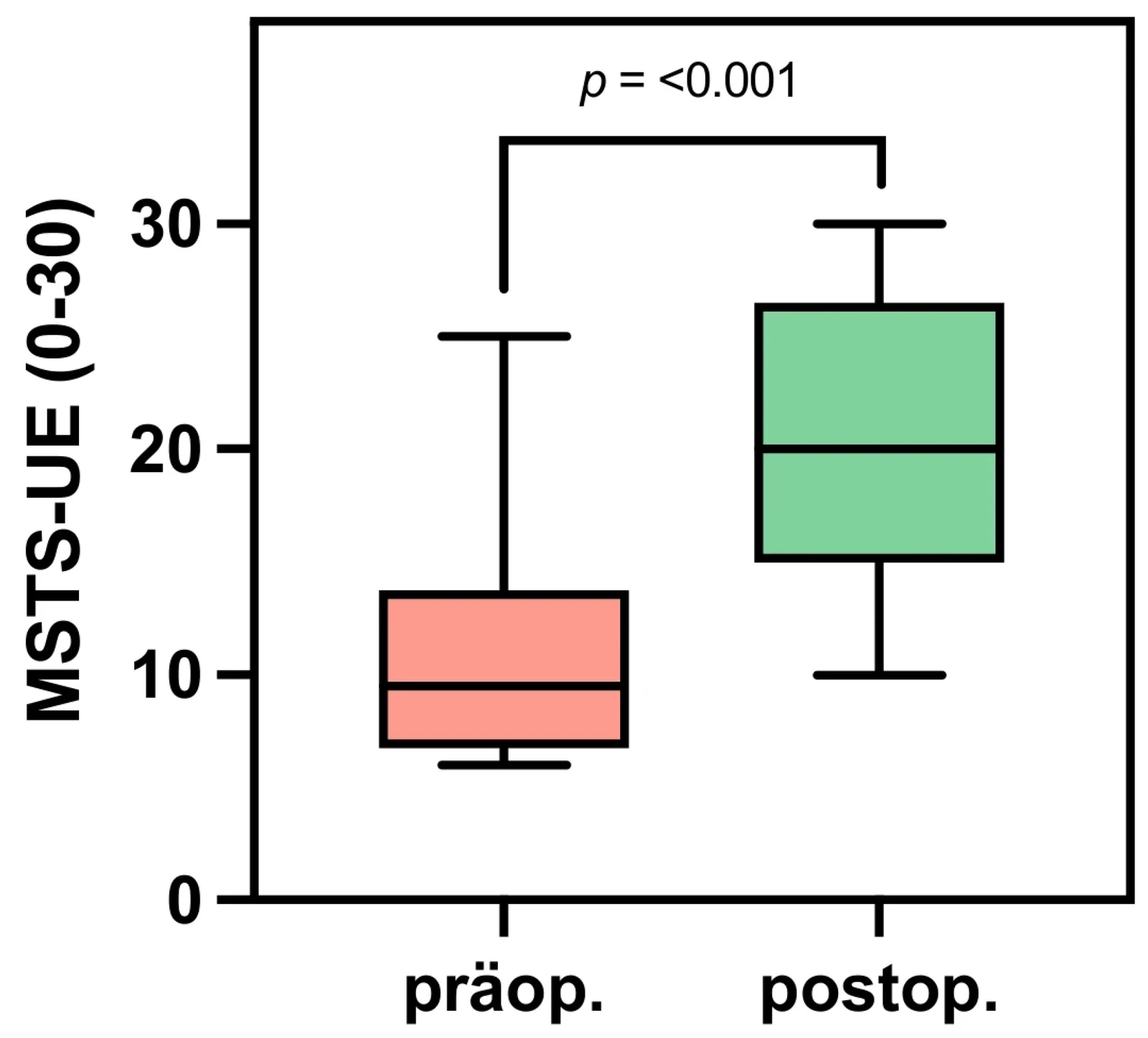
Comparison of preoperative and postoperative MSTS-UE values after photodynamic stabilization in patients with pathologic humerus fractures, paired *t*-test, *n* = 26.

**Figure 3:**
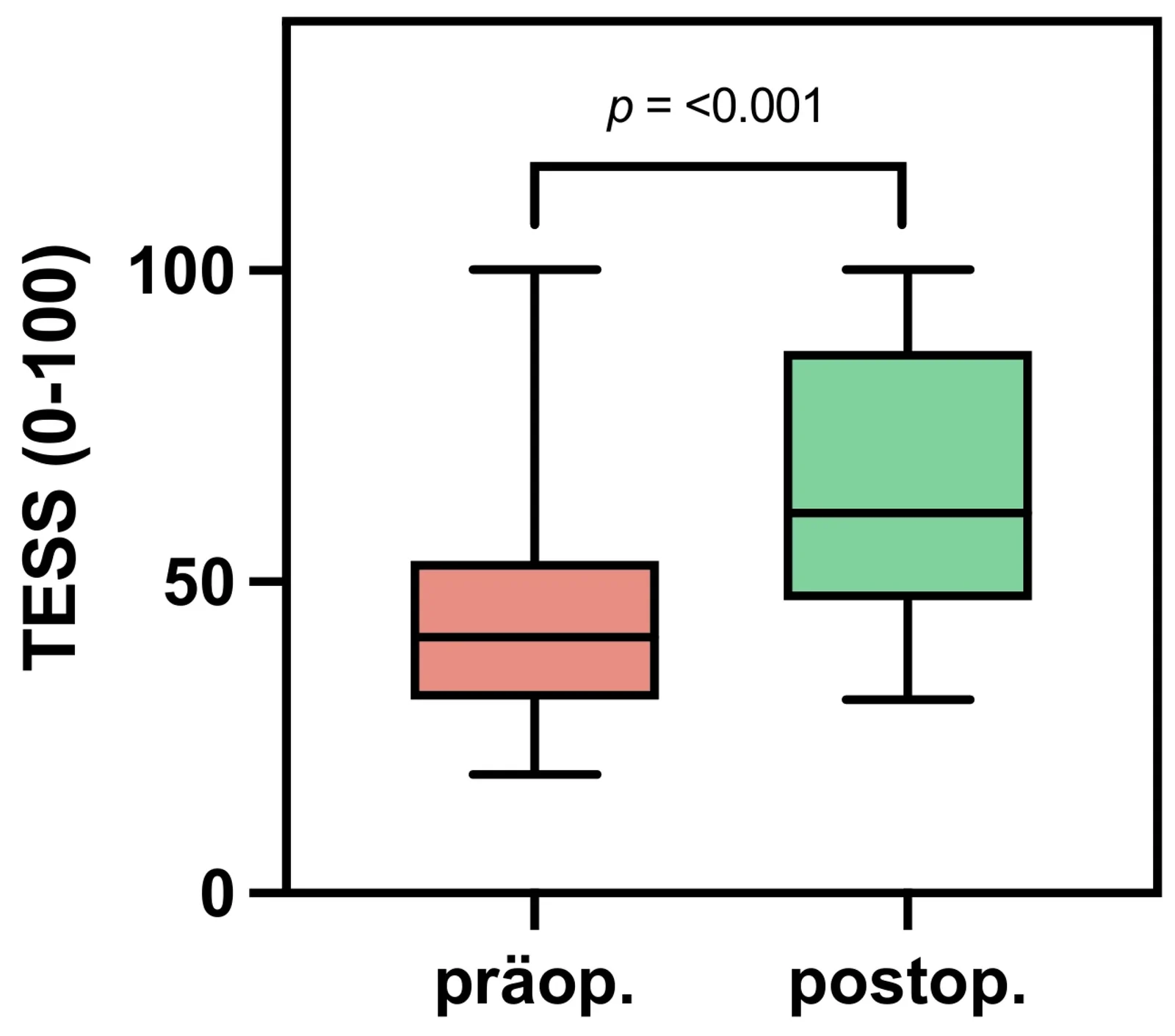
Comparison of preoperative and postoperative TESS values after photodynamic stabilization in patients with pathologic humerus fractures, paired *t*-test, *n* = 26.

**Table 1:** Overview of the characteristics of the four implant failure cases.

Table Implant Failures	Case 1	Case 2	Case 3	Case 4
Fracture status (impending vs. complete)	complete	complete	complete	complete
Anatomical site	diaphysis	proximal diaphysis	diaphysis	diaphysis
Extent of bone destruction	>2/3	2/3	1/3	2/3
Supplementary fixation during initial surgery	screw osteosynthesis	none	plate osteosynthesis	none
Time to failure (days)	9	75	409	62
Mechanism of failure	Henderson type III	Henderson type III	Henderson type III	Henderson type III
Revision treatment	plate osteosynthesis	plate osteosynthesis	plate/screw osteosynthesis	diaphyseal replacement

## Data Availability

The data underlying this study are not publicly available due to privacy and data protection regulations but can be made available from the corresponding author upon reasonable request and subject to institutional approval.

## References

[1] Kendal J.K., Heard B.J., Abbott A.G., Moorman S.W., Saini R., Puloski S.K.T., Monument M.J. (2022). Does surgical technique influence the burden of lung metastases in patients with pathologic long bone fractures?. BMC Musculoskelet. Disord..

[2] Amen T.B., Varady N.H., Birir A., Hayden B.L., Chen A.F. (2021). Morbidity and mortality of surgically treated pathologic humerus fractures compared to native humerus fractures. J. Shoulder Elb. Surg..

[3] Maurer F., Ambacher T., Volkmann R., Weller S. (1995). Pathologische Frakturen: Diagnostische und therapeutische Überlegungen sowie Behandlungsergebnisse. Langenbecks Arch. Chir..

[4] Kobryn A., Nian P., Baidya J., Li T.L., Maheshwari A.V. (2023). Intramedullary nailing with and without the use of bone cement for impending and pathologic fractures of the humerus in multiple myeloma and metastatic disease. Cancers.

[5] Coleman R.E. (2006). Clinical features of metastatic bone disease and risk of skeletal morbidity. Clin. Cancer Res..

[6] Cirstoiu C., Cretu B., Iordache S., Popa M., Serban B., Cursaru A. (2022). Surgical management options for long-bone metastasis. EFORT Open Rev..

[7] Biermann J.S., Holt G.E., Lewis V.O., Schwartz H.S., Yaszemski M.J. (2009). Metastatic bone disease: Diagnosis, evaluation, and treatment. J. Bone Jt. Surg. Am..

[8] Voskuil R.T., Mayerson J.L., Scharschmidt T.J. (2021). Management of metastatic disease of the upper extremity. J. Am. Acad. Orthop. Surg..

[9] Saad F., Lipton A., Cook R., Chen Y.M., Smith M., Coleman R. (2007). Pathologic fractures correlate with reduced survival in patients with malignant bone disease. Cancer.

[10] Chen B.K., Tai T.H., Lin S.H., Chen K.H., Huang Y.M., Chen C.Y. (2024). Intramedullary nail vs. plate fixation for pathological humeral shaft fracture: An updated narrative review and meta-analysis of surgery-related factors. J. Clin. Med..

[11] Choi E.S., Han I., Cho H.S., Park I.W., Park J.W., Kim H.S. (2016). Intramedullary nailing for pathological fractures of the proximal humerus with cement augmentation. Clin. Orthop. Surg..

[12] Ricard M.A.M., Stavropoulos N.A., Nooh A., Ste-Marie N., Goulding K., Turcotte R. (2021). Intramedullary nailing versus plate osteosynthesis for humeral shaft metastatic lesions. Cureus.

[13] Hoellwarth J.S., Weiss K., Goodman M., Heyl A., Hankins M.L., McGough R. (2020). Evaluating the reoperation rate and hardware durability of three stabilizing implants for 105 malignant pathologic humerus fractures. Injury.

[14] Yeung C.M., Bhashyam A.R., Groot O.Q., Merchan N., Newman E.T., Raskin K.A., Lozano-Calderón S.A. (2022). Comparison of carbon fibre and titanium intramedullary nails in orthopaedic oncology. Bone Jt. Open.

[15] Gausepohl T., Pennig D., Heck S., Gick S., Vegt P.A., Block J.E. (2017). Effective management of bone fractures with the IlluminOss^®^ photodynamic bone stabilization system: Initial clinical experience from the European Union registry. Orthop. Rev..

[16] Zoccali C., Attala D., Pugliese M., di Uccio A.S., Baldi J. (2021). The IlluminOss^®^ photodynamic bone stabilization system for pathological osteolyses and fractures of the humerus: Indications, advantages and limits in a series of 12 patients at 24 months of minimum follow-up. BMC Musculoskelet. Disord..

[17] Vegt P., Verbruggen J., Gausepohl T., Vroemen J.P., Schafer W., Pennig D., Muir J.M. (2018). The photodynamic bone stabilization system in the treatment of humerus fractures: A prospective clinical trial. Cureus.

[18] Zhu K.Y., McNassor R., Hecht C.J., Burkhart R.J., Nystrom L.M., Kamath A.F. (2024). Photodynamic bone stabilization for traumatic and pathologic fractures: A systematic review of utilization, complications, and patient-reported outcomes. Eur. J. Orthop. Surg. Traumatol..

[19] Nixon B., Stewart S., Crawford B., Temple T., Munera F., Jose J. (2024). Novel IlluminOss photodynamic bone stabilization system: Normal and post-operative complication imaging findings in the emergency setting. Emerg. Radiol..

[20] Enneking W.F., Dunham W., Gebhardt M.C., Malawar M., Pritchard D.J. (1993). A system for the functional evaluation of reconstructive procedures after surgical treatment of tumors of the musculoskeletal system. Clin. Orthop. Relat. Res..

[21] Davis A.M., Wright J.G., Williams J.I., Bombardier C., Griffin A., Bell R.S. (1996). Development of a measure of physical function for patients with bone and soft tissue sarcoma. Qual. Life Res..

[22] Hoban K.A., Downie S., Adamson D.J.A., MacLean J.G., Cool P., Jariwala A.C. (2022). Mirels’ score for upper limb metastatic lesions: Do we need a different cutoff for recommending prophylactic fixation?. JSES Int..

[23] Evans A.R., Bottros J., Grant W., Chen B.Y., Damron T.A. (2008). Mirels’ rating for humerus lesions is both reproducible and valid. Clin. Orthop. Relat. Res..

[24] Terek R.M. (2024). IlluminOss photodynamic bone stabilization system improves pain and function in the treatment of humeral metastatic disease. Bone Jt. J..

[25] Hu Y., Wu T., Li B., Huang Y., Huang C., Luo Y. (2022). Efficacy and safety evaluation of intramedullary nail and locking compression plate in the treatment of humeral shaft fractures: A systematic review and meta-analysis. Comput. Math. Methods Med..

[26] Griggers J.I., Alcantar S., Gonzalez M.R., Lozano-Calderon S.A. (2025). Postoperative complications of intramedullary nailing for impending and pathologic fractures of the humerus due to bone metastases—A systematic review of the literature. J. Surg. Oncol..

[27] Sørensen J.B., Klee M., Palshof T., Hansen H.H. (1993). Performance status assessment in cancer patients: An inter-observer variability study. Br. J. Cancer.

[28] Wu X.T., Zhou J.W., Pan L.C., Ge T. (2020). Clinical features and prognostic factors in patients with bone metastases from non-small cell lung cancer. J. Int. Med. Res..

[29] Arpornsuksant P., Morris C.D., Forsberg J.A., Levin A.S. (2022). What factors are associated with local metastatic lesion progression after intramedullary nail stabilization?. Clin. Orthop. Relat. Res..

[30] Al Farii H., McChesney G., Patel S.S., Satcher R., Frink S., Moon B., Lin P., Lewis V., Bird J.E. (2025). Device-related clinical outcomes of the Photodynamic Bone Stabilization System in the treatment of pathologic humerus fractures: A meta-analysis of the literature. Med. Res. Arch..

